# Entry Inhibition and Modulation of Pro-Inflammatory Immune Response Against Influenza A Virus by a Recombinant Truncated Surfactant Protein D

**DOI:** 10.3389/fimmu.2018.01586

**Published:** 2018-07-30

**Authors:** Mohammed N. Al-Ahdal, Valarmathy Murugaiah, Praveen M. Varghese, Suhair M. Abozaid, Iram Saba, Ahmed Ali Al-Qahtani, Ansar A. Pathan, Lubna Kouser, Béatrice Nal, Uday Kishore

**Affiliations:** ^1^Department of Infection and Immunity, King Faisal Specialist Hospital and Research Centre, Riyadh, Saudi Arabia; ^2^Biosciences, College of Health and Life Sciences, Brunel University London, Uxbridge, United Kingdom; ^3^Allergy & Clinical Immunology Inflammation, Repair and Development, Imperial College London, London, United Kingdom

**Keywords:** innate immunity, influenza A virus, surfactant protein D, pseudotyped lentiviral particles, inflammation

## Abstract

Surfactant protein D (SP-D) is expressed in the mucosal secretion of the lung and contributes to the innate host defense against a variety of pathogens, including influenza A virus (IAV). SP-D can inhibit hemagglutination and infectivity of IAV, in addition to reducing neuraminidase (NA) activity via its carbohydrate recognition domain (CRD) binding to carbohydrate patterns (N-linked mannosylated) on NA and hemagglutinin (HA) of IAV. Here, we demonstrate that a recombinant fragment of human SP-D (rfhSP-D), containing homotrimeric neck and CRD regions, acts as an entry inhibitor of IAV and downregulates M1 expression considerably in A549 cells challenged with IAV of H1N1 and H3N2 subtypes at 2 h treatment. In addition, rfhSP-D downregulated mRNA levels of TNF-α, IFN-α, IFN-β, IL-6, and RANTES, particularly during the initial stage of IAV infection of A549 cell line. rfhSP-D also interfered with IAV infection of Madin Darby canine kidney (MDCK) cells through HA binding. Furthermore, rfhSP-D was found to reduce luciferase reporter activity in MDCK cells transduced with H1+N1 pseudotyped lentiviral particles, where 50% of reduction was observed with 10 µg/ml rfhSP-D, suggestive of a critical role of rfhSP-D as an entry inhibitor against IAV infectivity. Multiplex cytokine array revealed that rfhSP-D treatment of IAV challenged A549 cells led to a dramatic suppression of key pro-inflammatory cytokines and chemokines. In the case of pH1N1, TNF-α, IFN-α, IL-10, IL-12 (p40), VEGF, GM-CSF, and eotaxin were considerably suppressed by rfhSP-D treatment at 24 h. However, these suppressive effects on IL-10, VEGF, eotaxin and IL-12 (p40) were not so evident in the case of H3N2 subtype, with the exception of TNF-α, IFN-α, and GM-CSF. These data seem to suggest that the extent of immunomodulatory effect of SP-D on host cells can vary considerably in a IAV subtype-specific manner. Thus, rfhSP-D treatment can downregulate pro-inflammatory milieu encouraged by IAV that otherwise causes aberrant inflammatory cell recruitment leading to cell death and lung damage.

## Introduction

The innate immune system is composed of both cellular and humoral players to encounter invading pathogens. It is also an important component in the initiation and modulation of the adaptive immunity. To distinguish self from non-self, the innate immune system has evolved to recognize pathogen-associated molecular patterns through a number of pattern recognition receptors, including toll like receptors and C-type lectin receptors. Collectins are collagenous lectins, representing a crucial group of calcium-dependent pattern recognition molecules present in pulmonary secretions and mammalian serum (1). They play a crucial role in the first line of defense against a diverse range of pathogens by interacting with specific glycoconjugates and lipid moieties present on the surface of microorganisms. A significant number of *in vitro and in vivo* studies have focused on the immunomodulatory functions of a lung collectin, human surfactant protein D (SP-D). SP-D is primarily organized into four regions: a cysteine-linked N-terminal region involved in multimerization, a triple-helical collagen region composed of Gly-X-Y repeats, an α-helical, coiled-coil trimerizing neck region, and the C-terminal carbohydrate recognition domains (CRDs) or C-type lectin domain (2). Human SP-D is primarily synthesized by alveolar type II and Clara cells, in addition to being present in several extra-pulmonary tissues. SP-D triggers a range of anti-microbial defense mechanisms, including agglutination/aggregation, phagocytosis, and direct growth inhibition (1). SP-D is also capable of controlling pulmonary inflammation including allergy and asthma, and thus, linking innate with adaptive immunity *via* modulation of dendritic cell maturation, and polarization of helper T cells (1).

The direct nature of interaction between SP-D and Influenza A Virus (IAV) has been reported (3, 4), which often results in virus neutralization and enhanced phagocytosis (5, 6). Anti-viral roles of SP-D during IAV infection have been well-documented, principally by Hartshorn group. IAV is an enveloped RNA virus and a member of Orthomyxoviridae family that possesses eight single-stranded RNA segments with negative orientation. These RNA segments can encode up to 13 viral proteins, including two surface glycoproteins, an ion channel protein, nucleocapsid protein, structural scaffolding protein, a tripartite polymerase complex, two non-structural proteins, and three non-essential proteins (7). IAV is subtyped based on their surface glycoproteins, such as hemagglutinin (HA) and neuraminidase (NA); to date, there are 19 HA and 9 NA protein subtypes that have been well established. Both HA and NA play an important role in the host range, viral replication, and pathogenicity (8). Among the three genera of influenza viruses reported, infection by IAV is the most common and severe in humans, swine, and avian species. It is also known to cause pandemic infections, being diverse in host specificity. IAV is considered as a major human respiratory pathogen following 1918 H1N1 influenza pandemic (Spanish Flu) (9), which is believed to have resulted in the zoonotic transmission of an avian virus to a human host and has rapidly dispersed (10).

Binding of IAV to target cells is mediated *via* the globular head of HA to sialic acid (SA) receptors present on the host cell surface (11, 12). IAV subtypes have adapted to human preferentially *via* binding with α (2–6) linkage of SA receptors (13). Following IAV–SA receptor interaction, virus particles are internalized *via* clathrin, resulting in clathrin-mediated endocytosis, or *via* caveolin/clathrin-independent mechanism (14, 15). Thus, acidic environment triggers M2 ion channel and transfers protons and potassium into the interior portion of the virion to dissociate M1 protein from the ribonucleoprotein (RNP) (16). Acidification also initiates HA-mediated conformational changes, which leads to viral fusion and RNPs release into the cytoplasm, resulting in viral transcription and replication. It is, therefore, suggested that SA and its linkage are crucial for the initiation of IAV infection of both epithelial and immune cells. Thus, inhibition of SA receptor binding or enzymatic switching of SA-mediated linkages can confer cell resistance, and/or alter susceptibility to IAV infection. Hence, cell surface SA is considered as an important primary receptor and determinant of IAV tropism, contributing to induction of immune responses as well as to viral pathogenesis.

It is crucial to understand the molecular mechanisms of host defense against IAV in order to design novel anti-IAV strategies. SP-D binding to HA leads to a direct inhibition of cellular infection by preventing HA–SA receptor interaction (4). SP-D has been shown to bind HA-mediated glycosylation sites, identified as β-type inhibitor of IAV. This interaction is calcium dependent, and binding of SP-D to NA inhibits the release of progeny virions from infected cells (17, 18). It has been reported that recombinant full-length porcine SP-D has a potent antiviral activity against a wide range of IAV by similar mechanisms, more than human SP-D due to structural differences, such as an additional loop in its CRD, an additional glycosylation site, and an additional cysteine in the collagen domain (19). In this study, we have used a well-characterized recombinant homotrimeric fragment of human SP-D comprising neck and CRD region (rfhSP-D), and examined its ability to act as an entry inhibitor of IAV and pseudotyped viral particles, and modulate subsequent immunological response *in vitro*.

## Materials and Methods

### Reagents

#### Viruses and Reagents

The A/England/2009 (pH1N1) and the A/HK/99 (H3N2) strains were gifted by Wendy Barclay from the Imperial College, London and Leo Poon from the University of Hong Kong, respectively. The plasmids used to produce the H1+N1 pseudotyped lentiviral particles were obtained from Addgene. The pHIV-Luciferase plasmid was a gift from Bryan Welm (Addgene plasmid # 21375); psPAX2 was a gift from Didier Trono (Addgene plasmid # 12260); and Vesicular Stomatitis Virus (VSV-G) was offered by Bob Weinberg (Addgene plasmid #8454). Monoclonal Anti-Influenza Virus H1 HA, A/California/04/2009 (H1N1)pdm09, Clone 5C12 (produced *in vitro*), NR-42019 and Polyclonal Anti-Influenza Virus H3 HA, A/Hong Kong/1/1968 (H3N2) (antiserum, Goat), NR-3118 were obtained from BEI Resources, NIAID, NIH, USA.

### Cell Culture

Adenocarcinomic human alveolar basal epithelial cells (A549), Madin Darby Canine Kidney (MDCK), and human embryonic kidney (HEK) 293T cell lines were cultured in Dulbecco’s Modified Eagle’s Medium (DMEM) (Sigma-Aldrich), supplemented with 10% v/v fetal bovine serum (FBS), 2 mM l-glutamine, 100 U/ml penicillin (Sigma-Aldrich), 100 µg/ml streptomycin (Sigma-Aldrich) and 1 mM sodium pyruvate (Sigma-Aldrich), and left to grow at 37°C in the presence of 5% v/v CO_2_ for approximately 3 days before passaging. Since these cells were adherent, they were detached using 2× Trypsin-EDTA (0.5%) (Fisher Scientific) for 10 min at 37°C. Cells were then centrifuged at 1,200 rpm for 5 min, followed by re-suspension in complete DMEM with FBS, penicillin, and streptomycin, as described above. To determine the cell count and viability, an equal volume of the cell suspension and Trypan Blue (0.4% w/v) (Fisher Scientific) solution were vortexed, followed by cell count using a hemocytometer with Neubauer rulings (Sigma-Aldrich). Cells were then re-suspended in complete DMEM for further use.

### Purification of IAV Subtypes

MDCK cells at 80–90% confluency were washed with sterile PBS twice before infection. Diluted pH1N1 (2 × 10^4^) or H3N2 (3.3 × 10^4^) (600 µl/flask) was transferred to the flask containing 20 ml of complete DMEM, and incubated at 37°C for 1 h. Unbound viruses were removed by washing three times with sterile PBS. 25 ml of infection medium [DMEM with 1% penicillin/streptomycin, 0.3% bovine serum albumin (BSA), and 1 µg/ml of l-1-Tosylamide-2-phenylethyl chloromethyl ketone (TPCK)—Trypsin] (Sigma-Aldrich) were added to the flasks, and incubated at 37°C for 3 days. The virus particles were then harvested *via* centrifugation of the infection medium at 3,000 × *g* at 4°C for 15 min. The supernatant obtained was centrifuged at 10,000 × *g* for 30 min at 4°C. 26 ml of supernatant was added slowly to new ultra-clear centrifuge tubes containing 30% w/v sucrose (8 ml/tube) (Sigma-Aldrich), and centrifuged at 25,000 × *g* at 4°C for 90 min. The upper phase of the medium and the sucrose phase were carefully removed; IAV particles at the bottom were re-suspended in 100 µl of sterile PBS. Virus suspension (15 µl) was subsequently analyzed by SDS-PAGE and ELISA.

### Tissue Culture Infectious Dose 50% (TCID_50_) Assay

Purified pH1N1 or H3N2 virus stocks were prepared with a starting dilution of 10^−2^ in DMEM and 146 µl of the diluted virus was added to all wells; uninfected MDCK cells were used as a control. 46 µl of pH1N1 or H3N2 was then serially diluted (1/2log_10_ up to 10^−7^) and incubated at 37°C for 1 h in a microtiter plate. 1 × 10^5^ MDCK cells, earlier trypsinised and re-suspended in 2× infection medium, were added to each well and incubated for 3 days at 37°C under 5% v/v CO_2_ until cytopathic effect (CPE) was observed. After 5 days, each well was observed under microscope, and the number of wells that were positive and negative for CPE at each dilution was recorded.

### Expression and Purification of a Recombinant Fragment of Human SP-D Containing Neck and CRD Regions

A recombinant fragment of human SP-D (rfhSP-D) was expressed under bacteriophage T7 promoter in *Escherichia coli* BL21 (λDE3) pLysS (Invitrogen), transformed with plasmid pUK-D1 containing cDNA sequences for the 8 Gly-X-Y repeats, neck and CRD regions of human SP-D, as described previously (20). Briefly, a primary inoculum of 25 ml bacterial culture was inoculated into 500 ml of LB containing 100 µg/ml ampicillin and 34 µg/ml chloramphenicol (Sigma-Aldrich), grown to OD_600_ of 0.6, and then induced with 0.5 mM isopropyl β-d-1-thiogalactopyranoside (IPTG) (Sigma-Aldrich) for 3 h. The bacterial cell pellet was re-suspended in lysis buffer (50 mM Tris–HCl pH7.5, 200 mM NaCl, 5 mM EDTA pH 8, 0.1% v/v Triton X-100, 0.1 mM phenyl-methyl-sulfonyl fluoride, 50 µg/ml lysozyme) and sonicated (five cycles, 30 s each). The sonicate was harvested at 12,000 × *g* for 30 min, followed by solubilization of inclusion bodies in refolding buffer (50 mM Tris–HCl pH 7.5, 100 mM NaCl, 10 mM 2-Mercaptoethanol) containing 8 M urea. The solubilized fraction was then dialyzed stepwise against refolding buffer containing 4 M, 2 M, 1 M, and no urea. The clear dialysate was loaded onto a maltose agarose column (5 ml; Sigma-Aldrich) and the bound rfhSP-D was eluted using 50 mM Tris–HCl, pH 7.5, 100 mM NaCl, and 10 mM EDTA. The eluted fractions were then passed through PierceTM High Capacity Endotoxin Removal Resin (Qiagen) to remove endotoxin. The endotoxin levels were measured *via* QCL-1000 Limulus amebocyte lysate system (Lonza), and found to be <5 pg/µg of rfhSP-D.

### Direct Binding ELISA

Maxisorp 96-well microtiter plates were coated with rfhSP-D (5, 2.5, 1.25, and 0.625 µg/well) in carbonate-bicarbonate buffer (CBC), pH 9.6, and incubated overnight at 4°C. After removing the CBC buffer, microtiter wells were washed with PBS three times, blocked with 2% w/v BSA in PBS for 2 h at 37°C, and then washed three times with PBST (PBS + 0.05% Tween 20). 20 µl of concentrated pH1N1, H3N2 virus (1.36 × 10^6^ pfu/ml), or purified recombinant HA (2.5 µg/ml) was diluted in 200 µl of PBS, 10 µl of diluted virus was added to each well, and incubated at room temperature (RT) for 2 h in buffer containing 5 mM CaCl_2_. VSV-G pseudotyped lentivirus was used as a negative control. The microtiter wells were washed with PBST three times and the binding was probed with primary antibody: monoclonal anti-influenza virus H1 (BEI-Resources) and polyclonal anti-influenza virus H3 (BEI-Resources) antibody (1:5,000 dilution in PBS) for 1 h at 37°C. The wells were washed again with PBST and incubated with anti-mouse IgG-Horseradish peroxidase (HRP)-conjugate (1:5,000) (Fisher Scientific) and Protein A-HRP-conjugate (Fisher Scientific) in PBS (100 µl/well), respectively, for 1 h at 37°C. Color was developed using 3,3′,5,5′-Tetramethylbenzidine (TMB) substrate (Sigma-Aldrich). The reaction was stopped using 2 N H_2_SO_4_ and the absorbance was read at 450 nm using iMark™ microplate absorbance reader (Bio-Rad).

### Far Western Blotting

rfhSP-D (5 µg) or 10 µl of concentrated pH1N1/H3N2 (1.36 × 10^6^ pfu/ml) were run separately on a 12% (w/v) SDS-PAGE, and then electrophoretically transferred onto a nitrocellulose membrane (320 mA for 2 h) in 1× transfer buffer (25 mM Tris–HCl pH 7.5, 190 mM glycine, and 20% methanol), followed by blocking overnight in 5% w/v dried milk powder in PBS (Sigma-Aldrich) at 4°C on a rotatory shaker. The membrane was then washed with PBST three times, 10 min each. For far western blotting, the nitrocellulose membrane was incubated with 5 µg/ml of rfhSP-D in PBS containing 5 mM CaCl_2_ for 1 h at RT and 1 h at 4°C. Following PBST wash, the membrane was incubated with primary antibodies, polyclonal rabbit anti-human SP-D, monoclonal anti-influenza virus H1 (BEI-Resources), or polyclonal anti-influenza virus H3 (BEI-Resources) in PBS (1:1,000) for 1 h at RT. Following washing, the membrane was probed with secondary antibodies: protein-A-HRP-conjugate, or rabbit anti-mouse IgG HRP conjugate (1:1,000) (Fisher Scientific) in PBS (100 µl/well) for 1 h at RT. After PBST wash, the blot was developed either using 3,3′-diaminobenzidine (DAB) or enhanced chemiluminescence substrate. For M1 detection, following 6 h incubation, both untreated (cells + virus) and treated samples (cells + virus + 10 µg/ml rfhSP-D) were run on the 12% (w/v) SDS-PAGE, and transferred onto a nitrocellulose membrane, as described above. The M1 expression was detected using anti-M1 monoclonal antibody (BEI-Resources).

### Cell-Binding Assay

A549 cells were seeded in microtiter wells using complete DMEM (1 × 10^5^ cells/well) and incubated overnight at 37°C. The wells were washed with PBS three times, and then rfhSP-D (10, 5, 2.5, and 1.25 µg/ml) was pre-incubated with pH1N1 or H3N2 virus (1.36 × 10^6^ pfu/ml) diluted in 200 µl of PBS + 5 mM CaCl_2_; 10 µl of diluted virus was added to the corresponding wells, and incubated at RT for 2 h. Maltose-binding protein (MBP) was used as a negative control. The microtiter wells were then washed with PBS three times, and fixed with 4% paraformaldehyde (Fisher Scientific) for 10 min at RT. The wells were washed again with PBS three times, and blocked with 2% w/v BSA in PBS for 2 h at 37°C. Monoclonal anti-influenza virus H1 (BEI-Resources) and polyclonal anti-influenza virus H3 (BEI-Resources) in PBS (1:5,000) were added to each well and incubated for 1 h at 37°C. After washing with PBST three times, the corresponding wells were probed with goat anti-mouse IgG-HRP-conjugate (Thermo-Fisher), or Protein A-HRP conjugate (1:5,000) in PBS for 1 h at 37°C. The wells were washed again with PBST three times and the color was developed using TMB substrate. The reaction was stopped using 2 M H_2_SO_4_, followed by absorbance reading at 450 nm.

### Titration Assay

Maxisorp 96-well plates were coated with 0.01% collagen (Sigma-Aldrich) and incubated at RT for 3 h. After removing the excess collagen, the wells were washed with PBS twice. 75,000 A549 cells were seeded and grown overnight at 37°C in the presence of 5% v/v CO_2_, until 75–80% confluency. Cells were washed with 1× PBS twice, pH1N1 or H3N2 virus (MOI of 1) diluted in pure DMEM with 10 µg/ml rfhSP-D was added to cells, respectively. The plates were incubated at 37°C for 1 h. The wells were then washed with PBS twice and 200 µl of infection medium was added to the cells, and incubated for 24 h at 37°C with 5% v/v CO_2_. The media of the infected cells in the presence or absence of rfhSP-D was collected and virus titer was estimated by TCID_50_.

### Infection Assay Using pH1N1 and H3N2

A549 cells were cultured in complete DMEM with usual supplements at 37°C in CO_2_ incubator until about 70–80% confluence. Cells, washed with PBS twice, trypsinised, and adjusted to 5 × 10^5^ cells in 12-well plates (Fisher Scientific), were left to adhere overnight at 37°C in serum-free complete DMEM. Cells were washed in PBS before the addition of rfhSP-D (10 µg/well) in pure DMEM containing 5 mM CaCl_2_ with MOI 1 of pH1N1 or H3N2 virus (1 h at RT and 1 h at 4°C). The pre-incubated virus and protein mix was then added onto the cells in a circular motion and incubated at 37°C for 1 h in DMEM only. Medium containing unabsorbed virus and rfhSP-D protein was removed, cells were washed with PBS twice, infection medium was added, and then left to incubate 2 and 6 h. The infected cells were detached by scrapping with a sterile cell scrapper, centrifuged at 1,500 × *g* for 3 min, and frozen at −80°C until further analysis *via* qPCR.

### Real-Time Quantitative PCR Analysis

The infected A549 cells were lysed using a lysis solution (50 mM Tris–HCl pH 7.5, 200 mM NaCl, 5 mM EDTA pH 8, 0.1% v/v Triton X-100). Total RNA was extracted using RNase Mini Kit (Qiagen). Contaminating DNA was removed by DNase I treatment, followed by heat-inactivation at 70°C of DNase I and RNase. A260 nm was used to quantify the amount of RNA using NanoDrop 2000/2000c (Sigma-Aldrich), and the RNA purity was assessed using A260/A280 ratio between 1.8 and 2.1. The isolated RNA was then converted into cDNA using SuperScript II Reverse Transcriptase (Thermo-Fisher Scientific). Oligo-dT primers were added to initiate cDNA synthesis and to avoid labeling of the rRNA and tRNA. cDNA was synthesized using high capacity RNA to cDNA Kit (Thermo-Fisher Scientific) using 1–2 µg of total RNA. Primer sequences were designed for specificity using the Primer-BLAST software (Basic Local Alignment Search Tool) (http://blast.ncbi.nlm.nih.gov/Blast.cgi) (Table 1). The qRT-PCR was performed using the Light Cycler system (Applied Biosciences). The amplification program used was at 95°C for 5 min, followed by 45 cycles of 95°C for 10 s, 60°C for 10 s, and 72°C for 10 s. The specificity of the assay was established by melting-curve analysis.

**Table 1:** Target genes, forward primers, and reverse primers used for qPCR.

Target	Forward primer	Reverse primer
18S	5′-ATGGCCGTTC TTAGTTGGTG-3′	5′-CGCTGAGCC AGTCAGTGTAG-3′

IL-6	5′-GAAAGCAGCA AAGAGGCACT-3′	5′-TTTCACCAGG CAAGTCTCCT-3′

IL-12	5′-AACTTGCAGC TGAAGCCATT-3′	5′-GACCTGAACG CAGAATGTCA-3′

TNF-α	5′-AGCCCATGTT GTAGCAAACC-3′	5′-TGAGGTACAG GCCCTCTGAT-3′

M1	5′AAACATATGTCTGATAAC GAAGGAGAACAGTTCTT-3′	5′GCTGAATTCTACCT CATGGTCTTCTTGA-3′

RANTES	5′-GCGGGTACCAT GAAGATCTCTG-3′	5′-GGGTCAGAATC AAGAAACCCTC-3′

IFN-α	5′-TTT CTC CTG CC T GAA GGA CAG-3′	5′-GCT CAT GAT TTC TGC TCT GAC A-3′

IFN-β	5′-AAA GAA GCA G CA ATT TTC AGC-3′	5′-CCT TGG CCT TCA GGT AAT GCA-3′

### Multiplex Cytokine Array Analysis

Supernatant from A549 cells, incubated with IAV with or without rfhSP-D for 24 h were collected for measuring secreted cytokines [TNF-α, IL-6, IL-10, IL-1α, interferon (IFN)-α, and IL-12p40], chemokine (eotaxin) and growth factors (GM-CSF and VEGF). The analytes were measured using MagPixMilliplex kit (EMD Millipore). 25 µl of assay buffer was added to each well of a 96-well plate, followed by addition of 25 µl of standard, control, or supernatant from A549 cells infected with pH1N1 or H3N2 (with or without rfhSP-D). 25 µl of magnetic beads, coupled to analytes, were added to each well, and incubated for 18 h at 4°C. The plate was washed with the assay buffer and 25 µl of detection antibodies were incubated with the beads for 1 h at RT. 25 µl of Streptavidin–Phycoerythrin was then added to each well and incubated for 30 min at RT. Following a washing step, 150 µl of sheath fluid was added to each well and the plate was read using the Luminex Magpix instrument. Assays were conducted in duplicate.

### Production of H1+N1 Pseudotyped Lentiviral Particles

HEK293T cells were co-transfected with 20 µg of pcDNA3.1-swineH1-flag (H1 from swine H1N1 A/California/04/09) (Invitrogen), pcDNA3.1-swine N1-flag (N1 from swine H1N1 A/California/04/09) (Invitrogen), pHIV-Luciferase backbone (Addgene), which carries a modified proviral HIV-1 genome with *env* deleted and designed to express the firefly luciferase reporter, and psPAX2 (Addgene). psPAX2 is a second-generation lentiviral packaging plasmid and can be used with second or third generation lentiviral vectors and envelope expressing plasmid. VSV-G lentivirus was produced in a similar way as described above, without H1+N1 plasmids. Supernatant containing the released H1+N1 pseudotyped and VSV-G lentiviral particles were harvested at 24 and 48 h and centrifuged at 5,000 × *g* for 10 min to remove any debris, and concentrated *via* ultra-centrifugation. The transfected HEK293T cells were lysed using lysis buffer (50 mM Tris–HCl pH 7.5, 200 mM NaCl, 5 mM EDTA, 0.1% v/v Triton X-100). The filtered supernatant and the cell lysate were analyzed *via* western blotting and luciferase reporter activity assay.

### Luciferase Reporter Activity Assay

MDCK cells were cultured in supplemented DMEM as described earlier, until about 70–80% confluency. The harvested H1+N1 pseudotyped particles at 24 and 48 h were used to perform luciferase reporter activity using luciferase one-step assay kit (Thermo Scientific). rfhSP-D (5 and 10 µg/ml) was used to determine its effect on the luciferase reporter activity; cells only, and cells + H1+N1 particles were used as controls. Readings were measured using a GloMax 96 Microplate Luminometer (Promega).

### Statistical Analysis

Graphs were generated using GraphPad Prism 6.0 software and the statistical analysis was performed using a two-way ANOVA test. Significant values were considered based on *p < 0.1, **p < 0.05, ***p < 0.01, and ****p < 0.001 between treated and untreated conditions. Error bars show the SD or SEM, as indicated in the figure legends.

## Results

### rfhSP-D Binds Directly to IAV Subtypes

**Figure 1:**
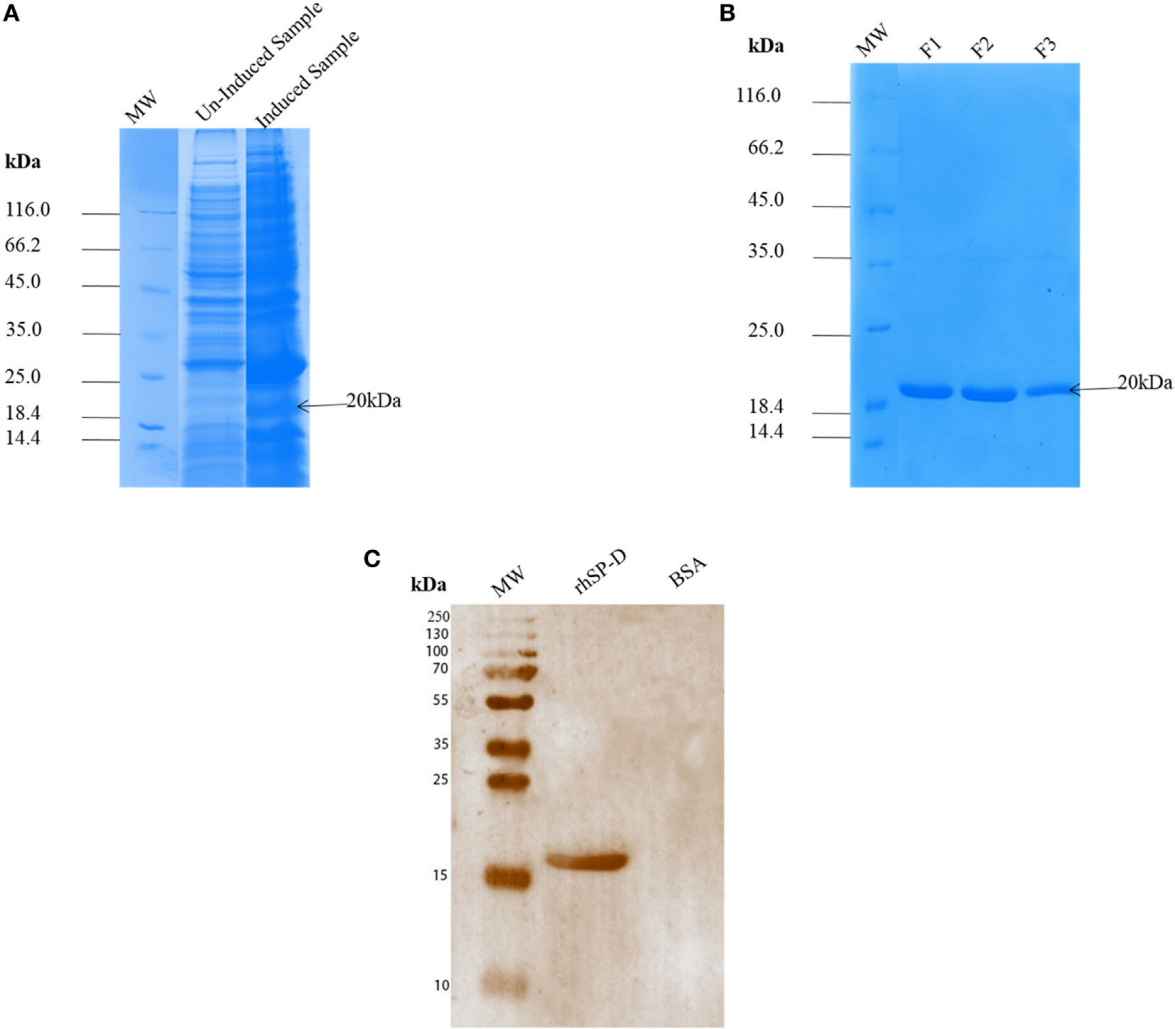
SDS-PAGE (12% v/v) under reducing conditions showing expression and purification of a recombinant surfactant protein D (rfhSP-D). The neck and carbohydrate recognition domain regions were expressed in *Escherichia coli* BL21 (λDE3) pLysS. **(A)** Following induction with 0.5 mM IPTG, a ~20 kDa band appeared being overexpressed compared to uninduced sample. Following denaturation–renaturation cycle, the rfhSP-D was purified on an affinity column to homogeneity after elution with EDTA as fractions F1, F2 and F3 **(B)**. A rabbit polyclonal antibody raised against full-length SP-D purified from human bronchoalveolar lavage **(C)** recognized the purified rfhSP-D, but not BSA that was used as a negative control protein.

**Figure 2:**
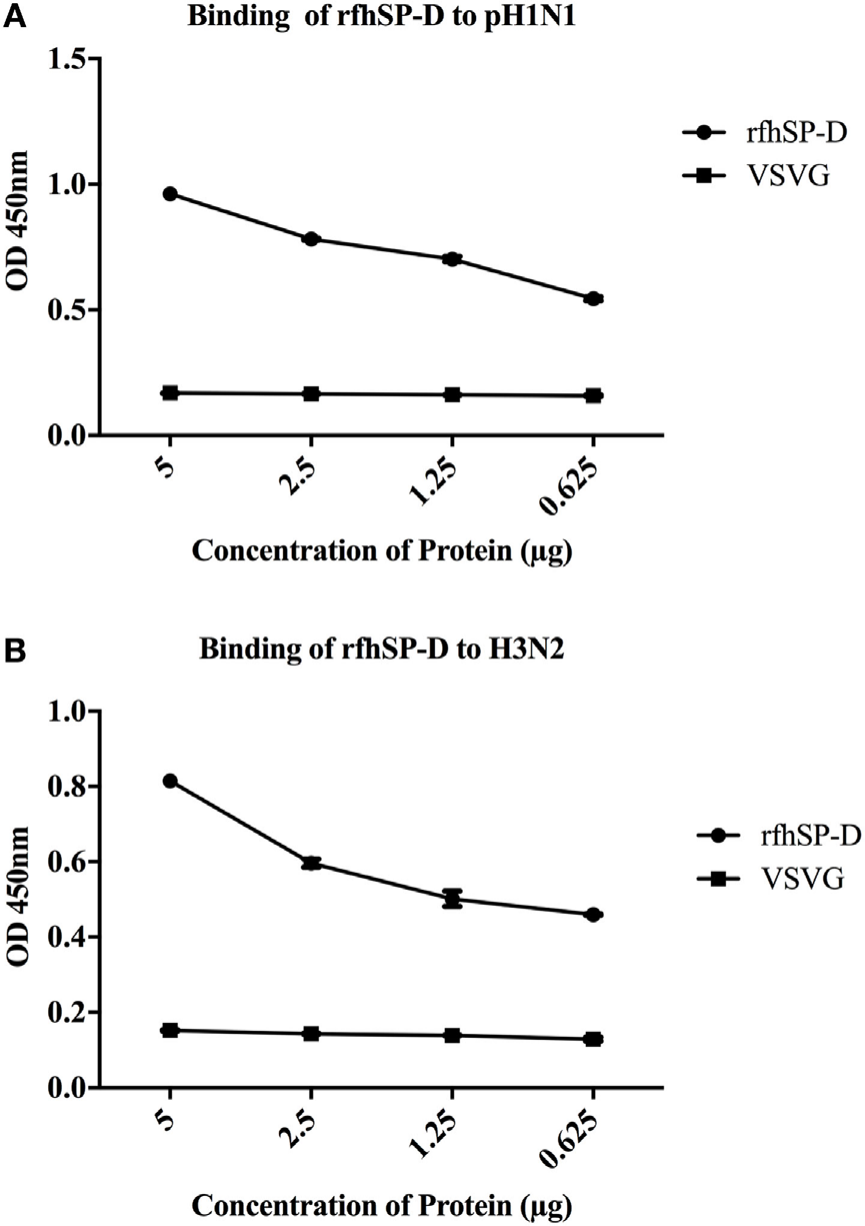
ELISA to show binding of rfhSP-D to **(A)** pH1N1 and **(B)** H3N2: microtiter wells were coated with different concentrations of rfhSP-D (5, 2.5, 1.25, and 0.625 µg/ml). 20 µl of concentrated pH1N1 or H3N2 virus (1.36 × 10^6^ pfu/ml) was diluted in 200 µl of PBS + 5 mM CaCl_2_ and 10 µl of diluted virus was added to all the wells, and probed with either monoclonal anti-influenza virus H1 or polyclonal anti-influenza virus H3 antibody. VSV-G pseudotyped lentivirus was used as a negative RNA virus control. The data were expressed as mean of three independent experiments done in triplicates ± SEM.

**Figure 3:**
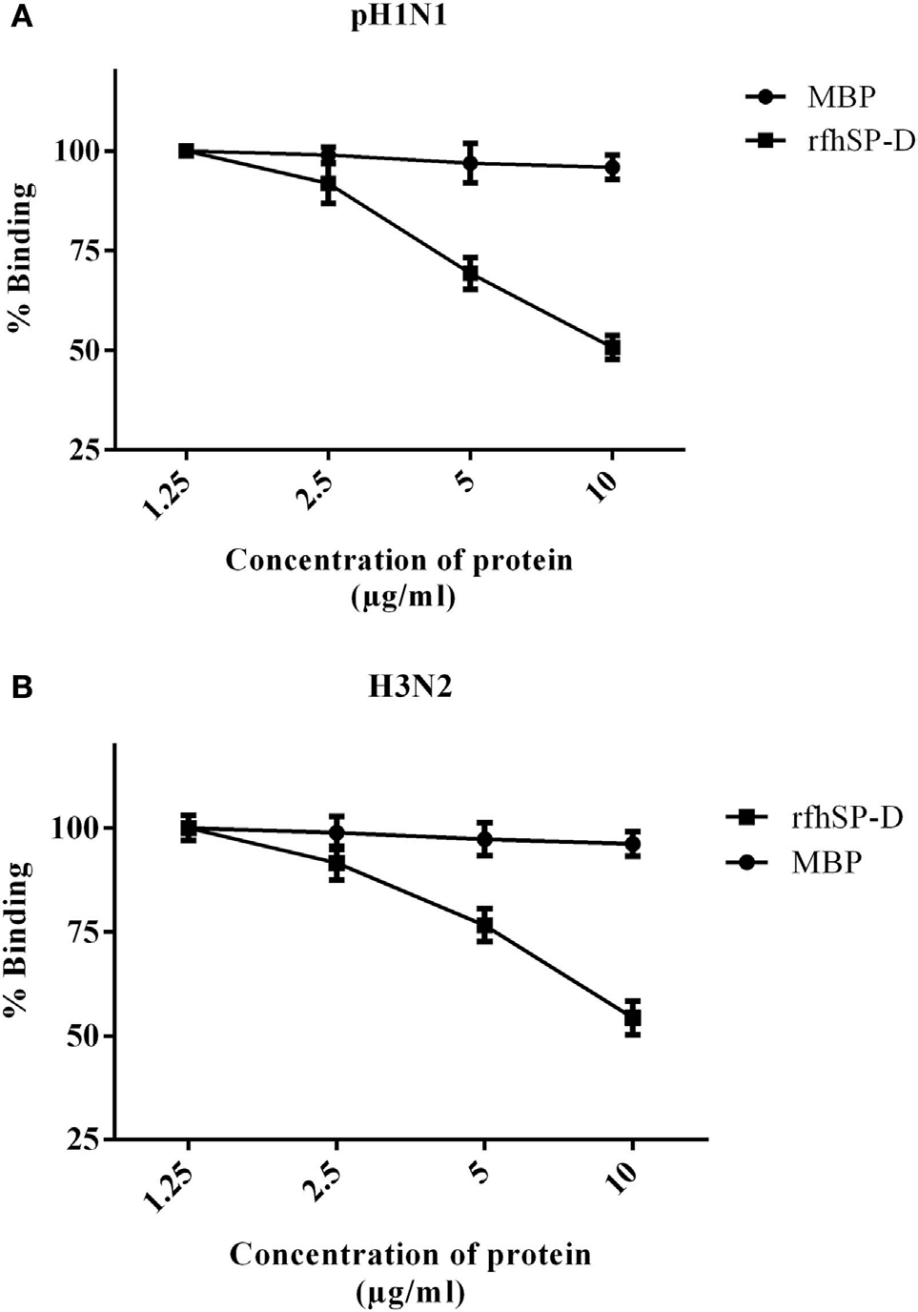
Cell-binding assay to show binding of **(A)** pH1N1 and **(B)** H3N2 pre-incubated with rfhSP-D to A549 cells. Microtiter wells were coated with A549 cells (1 × 10^5^ cells/ml) and incubated overnight at 37°C. Varied concentrations of pre-incubated rfhSP-D (10, 5, 2.5, and 1.25 µg/ml) with pH1N1 and H3N2 virus were added to the corresponding wells, followed by incubation at room temperature for 1–2 h. After fixing the cells with 4% paraformaldehyde solution, monoclonal anti-influenza virus H1, or polyclonal anti-influenza virus H3 were added to corresponding well. Maltose-binding protein (MBP) was used as a negative control protein. The data were expressed as mean of three independent experiments done in triplicates ± SEM.

*Escherichia coli* BL21 (λDE3) pLysS containing pUK-D1 construct (20) expressed a ~20 kDa protein following IPTG induction, compared to the un-induced bacterial cells (Figure 1A). The overexpressed insoluble rfhSP-D as inclusion bodies was refolded *via* denaturation and renaturation cycle. The soluble rfhSP-D fractions were affinity purified using maltose–agarose column, which appeared as a single band on 12% SDS-PAGE (v/v) under reducing condition (Figure 1B). The immunoreactivity of purified rfhSP-D was confirmed *via* western blotting using rabbit polyclonal anti-human SP-D antibody that was raised against native human SP-D purified from lung lavage of alveolar proteinosis patients (Figure 1C). The ability of pH1N1 and H3N2 subtypes to bind microtiter-coated rfhSP-D was examined *via* ELISA. As shown in Figure 2, rfhSP-D bound both IAV subtypes in a dose- and calcium-dependent manner. VSV-G pseudotyped lentivirus was used as a negative control RNA virus, where no significant binding was seen with all rfhSP-D concentrations tested. For cell-binding assay, A549 cells were challenged with purified pH1N1 or H3N2 pre-incubated with a range of rfhSP-D concentrations (Figure 3). The maximum inhibition (50%) of cell binding was seen at 10 µg/ml. MBP was used as a negative control protein.

### rfhSP-D Binds to HA and Restricts Replication of IAV in A549 Cells

**Figure 4:**
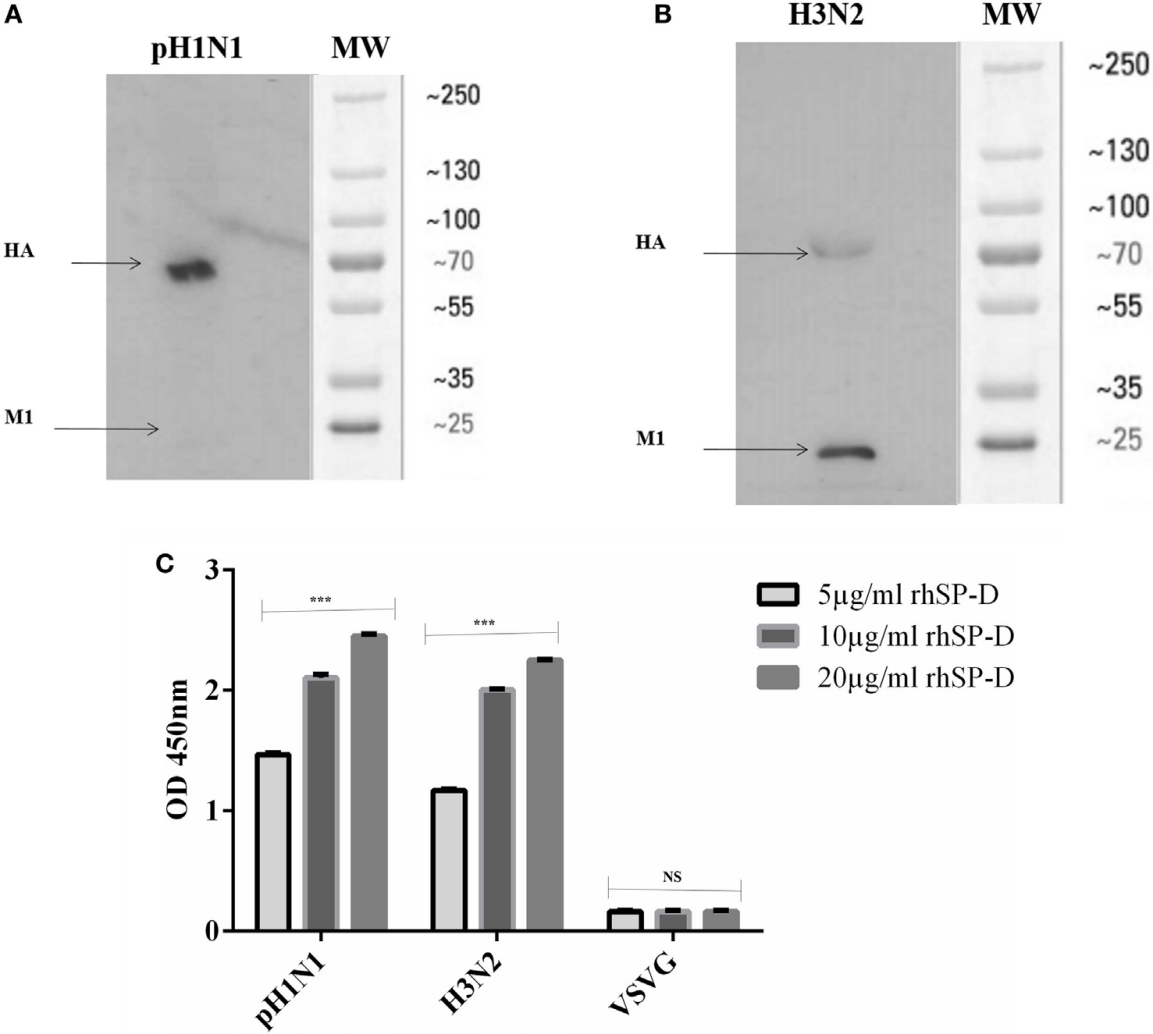
Far western blot analysis to show rfhSP-D binding to purified **(A)** pH1N1 and **(B)** H3N2: 10 μl of concentrated virus (1.36 × 10^6^ pfu/ml) was first run on the SDS-PAGE under reducing conditions, and then transferred onto a nitrocellulose membrane and incubated with 5 µg of rfhSP-D. The membrane was probed with anti-rabbit SP-D polyclonal antibodies. rfhSP-D bound to HA (70 kDa) and M1 (27 kDa) in the case of both pH1N1 and H3N2 subtypes. **(C)** ELISA to show the binding of rfhSP-D to purified recombinant hemagglutinin (HA) (μg/ml). VSV-G was used as a negative control. The data were expressed as mean of three independent experiments carried out in triplicates ± SEM. Significance was determined using the unpaired one-way ANOVA test (***p < 0.0001) (*n* = 3).

**Figure 5:**
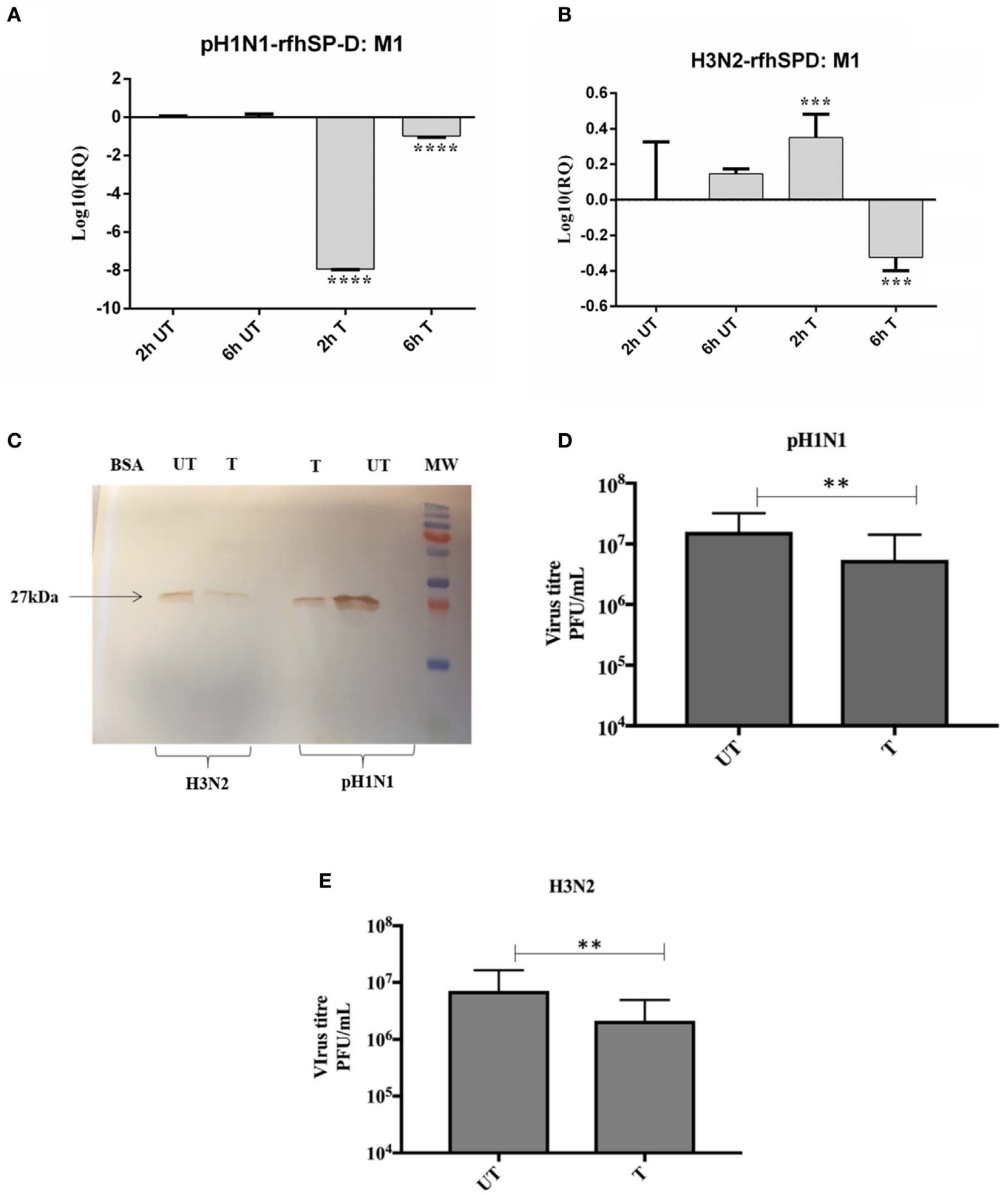
rfhSP-D restricts replication of **(A)** pH1N1 and **(B)** H3N2 in target human A549 cells. M1 expression of both pH1N1 and H3N2 influenza A virus (IAV) (MOI 1) after infection of A549 cells at differential time points at 2 and 6 h. A549 cells were incubated either with pre-incubated pH1N1 and H3N2 with (10 µg) or without purified rfhSP-D. Cell pellets were harvested at 2 and 6 h to analyze the M1 expression of IAV. Cells were lysed, and purified RNA extracted was converted into cDNA. Infection was measured *via* qRT-PCR using M1 primers and 18S was used as an endogenous control. Results shown are normalized to M1 levels at 2 h untreated. Significance was determined using the unpaired one-way ANOVA test (**p < 0.01, ***p < 0.001, and ****p < 0.0001) (*n* = 3). **(C)** Western blotting to shown M1 expression in both untreated (cells + virus) and treated (cells + virus + 10 µg/ml rfhSP-D) following 6 h incubation. Titration assay to show the anti-IAV activity of rfhSP-D (10 µg/ml), using both pH1N1 **(D)** and H3N2 **(E)** subtypes. A549 cells were infected with pH1N1/H3N2 (MOI 1) for 24 h. Then, the supernatants were collected and virus titers measured using a TCID50 assay. Treatment with rfhSP-D reduced viral titers by approximately 40%, suggesting that rfhSP-D acts as an entry inhibitor.

Previous studies have shown that SP-D binds to the glycosylation site of HA1 domain on IAV (18). Far western blotting revealed that rfhSP-D bound to HA (70 kDa) and M1 (27 kDa) of pH1N1 (Figure 4A) and H3N2 (Figure 4B) subtypes. As shown in Figure 4C, rfhSP-D was able to bind purified recombinant HA protein in a concentration-dependent manner. The binding of rfhSP-D may inhibit cellular viral infection by restricting the interaction of HA with SA containing receptors, and HA-mediated fusion in endosomes. The interaction between rfhSP-D and HA appears to offer another dimension at which rfhSP-D may suppress target cell infection and intracellular replication. The mechanism of direct inhibition of IAV by rfhSP-D was thus investigated *via* infection assay. A549 cells infected with pH1N1 and H3N2 revealed an upregulation of M1 expression at 2 and 6 h time points (Figure 5). However, A549 cells, pre-treated with rfhSP-D showed down-regulation of viral M1 expression when compared to untreated cells challenged with virus (Figure 5). The downregulation of M1 expression due to rfhSP-D pre-incubation was more effective in the case of pH1N1 compared to H3N2, where −8 log_10_ fold downregulation was seen at 2 h (Figure 5A). This was validated *via* western blotting, where a low M1 expression was detected in rfhSP-D (10 µg/ml) treated sample following 6 h incubation, when compared to untreated samples (cells + virus) (Figure 5C). Furthermore, anti-IAV activity of rfhSP-D was confirmed *via* virus titration assay (Figures 5D,E). Approximately 40% titer reduction was seen in 10 µg rhfSP-D treated cells compared to untreated samples, suggesting the ability of rfhSP-D to act as an entry inhibitor. Differential inhibitory effects of rfhSP-D on IAV subtypes may reflect on the glycosylation of the HA protein of IAV, suggesting a correlation between HA-glycan attachment and susceptibility of IAV strains to inhibition by rfhSP-D that involves specific interaction sites on HA.

### rfhSP-D Modulates Pro-Inflammatory Cytokine/Chemokine Immune Responses Following Virus Challenge to A549 Cells

**Figure 6:**
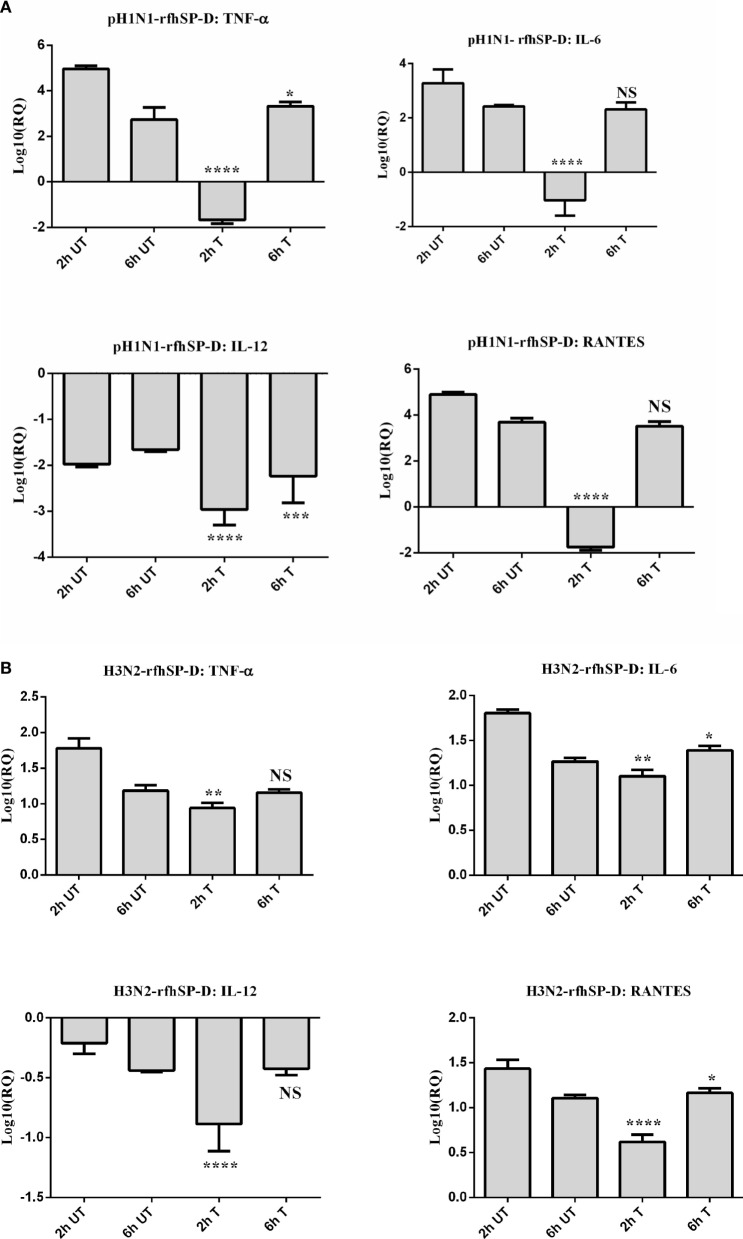

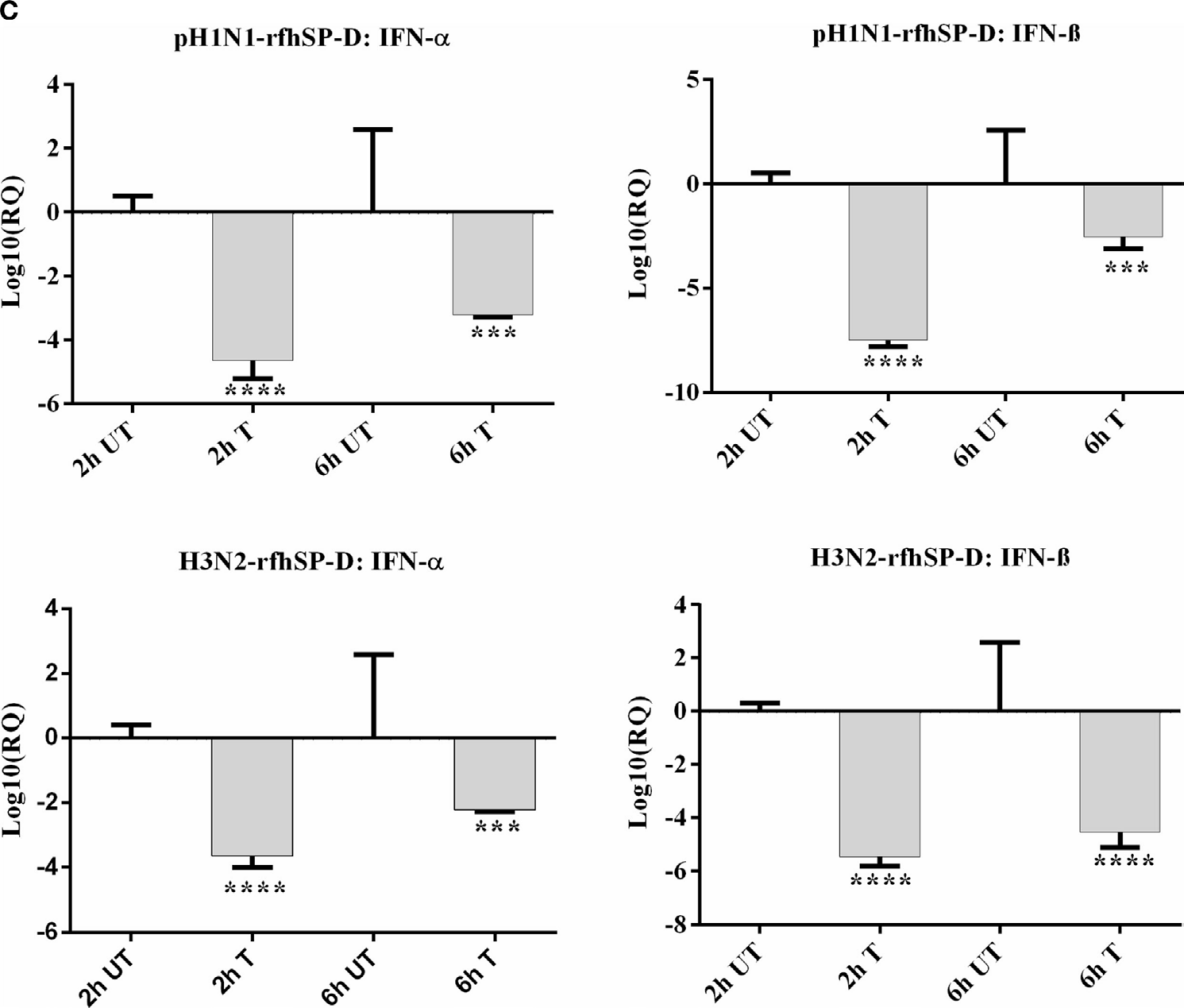
Differential mRNA expression profile of A549 cells challenged with pre-incubated **(A)** pH1N1, **(B)** H3N2 with rfhSP-D, and **(C)** expression levels of type I interferon (IFN) subtypes in both untreated and treated samples. The expression levels of cytokines and chemokine were measured using qRT-PCR and the data were normalized *via* 18S rRNA expression as a control. The relative expression (RQ) was calculated by using cells only time point as the calibrator. The RQ value was calculated using the formula: RQ = 2^−ΔΔCt^. Assays were conducted in triplicates and error bars represents ± SEM. Significance was determined using the unpaired one-way ANOVA test (*p < 0.05, **p < 0.01, ***p < 0.001, and ****p < 0.0001) (*n* = 3).

The qPCR analysis revealed that there was an upregulation of pro-inflammatory cytokines TNF-α and IL-6 by H3N2 strain, which were brought down slightly by rfhSP-D at 2 h (Figure 6A). However, both TNF-α and IL-6 in the case of pH1N1 were found to be downregulated considerably by rfhSP-D at 2 h, which gradually recovered by 6 h (Figure 6B). IL-6, which is crucial for the resolution of IAV infection, acts by inducing neutrophil mediated viral clearance. An elevated level of IL-6 in lung and serum has been reported in patients infected with pH1N1 (21). TNF-α and IL-6 are the key contributors to IAV-mediated respiratory diseases and acute lung injury. By contrast, there was a broad level of downregulation of IL-12 in the case of both IAV subtypes incubated with rfhSP-D, suggesting a likely reduction of Th1 response and suppression of IFN-γ production by CD4^+^ T cells. Suppressed transcript level of RANTES (1 log_10_ fold) by rfhSP-D was observed at 2 h treatment in the case of pH1N1. However, in the case of H3N2 strain, RANTES was downregulated by 0.5-fold (log_10_) (Figure 6B) at 2 h following treatment with rfhSP-D compared to untreated A549 cells. Furthermore, suppression of IFN-α and IFN-β were also seen with rfhSP-D treatment at both 2 and 6 h time points (Figure 6C). Both of these type I IFN cytokines play a crucial anti-viral role against IAV, and determine the rate of viral replication in the initial stages of infection. Suppression of type I IFN levels suggests the ability of rfhSP-D to reduce the rate of viral replication, thereby reducing the levels of INF produced by the innate immune system.

### Multiplex Cytokine Array Analysis Reveals a Differential Ability of rfhSP-D to Downregulate Pro-Inflammatory Cytokines and Chemokines

**Figure 7:**
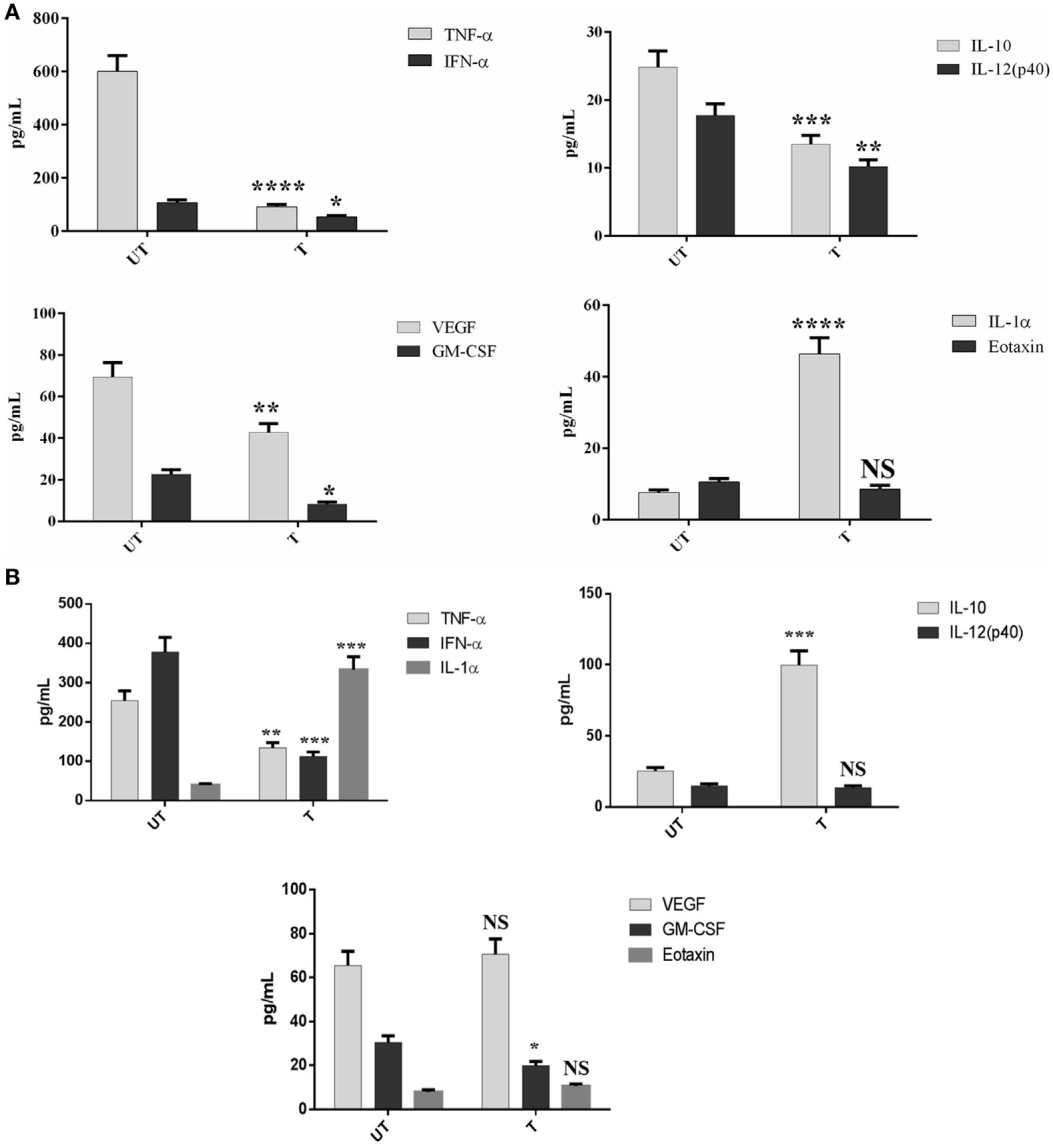
Multiplex cytokine array analysis of supernatants that were collected at 24 h time point. A549 cells were infected with pH1N1 **(A)** and H3N2 **(B)**, treated with 10 µg/ml of rfhSP-D. Cytokines (TNF-α, IL-6, IL-10, IL-1α, IFN-α, and IL-12p40), chemokine (eotaxin), and growth factors (GM-CSF and VEGF) were measured using a commercially available MagPix Milliplex kit (EMD Millipore). Assays were conducted in triplicates and error bars represent ± SEM (*n* = 3); significance was determined using unpaired one-way ANOVA test (*p < 0.05, **p < 0.01, ***p < 0.001 and ****p < 0.0001).

To assess secretion of cytokines, chemokines, and growth factors over a period of 24 h post rfhSP-D treatment, a multiplex cytokine array was performed using supernatants of the IAV challenged and rfhSP-D treated A549 cells. rfhSP-D induced a dramatic suppression of some of the key pro-inflammatory cytokines and chemokines in the virus infected A549 cells. In the case of pH1N1, TNF-α, IFN-α, IL-10, IL-12 (p40), VEGF, GM-CSF, and eotaxin were considerably suppressed by rfhSP-D treatment at 24 h (Figure 7A). However, these suppressive effects on IL-10, VEGF, eotaxin, and IL-12 (p40) were not so evident in the case of H3N2 subtype, with the exception of TNF-α, IFN-α, and GM-CSF (Figure 7). These data seem to suggest that the extent of immunomodulatory effect of rfhSP-D on host cells can vary considerably in a IAV subtype-specific manner.

### rfhSP-D Binds to H1+N1 Pseudotyped Lentivirus and Reduces Luciferase Reporter Activity

**Figure 8:**
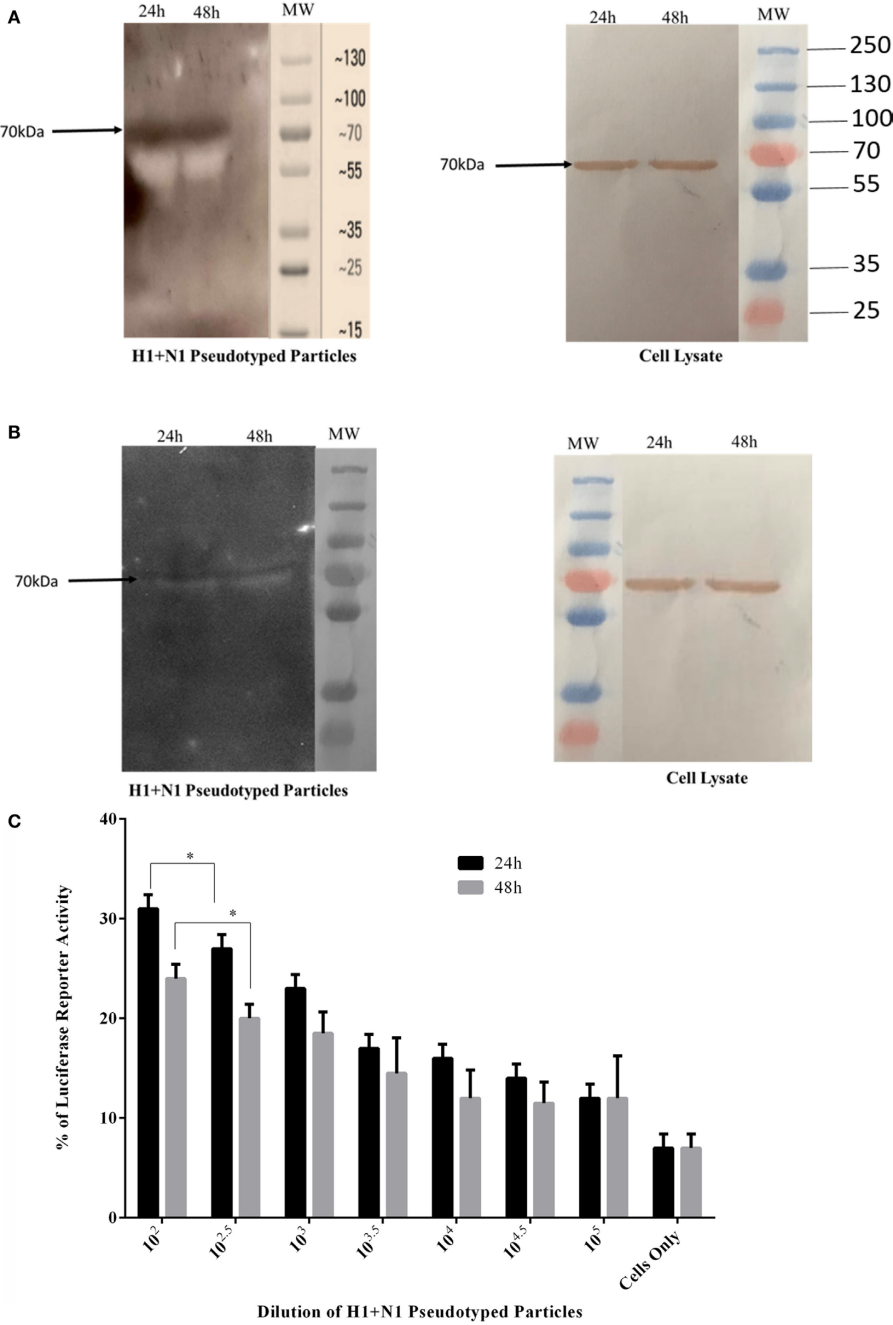

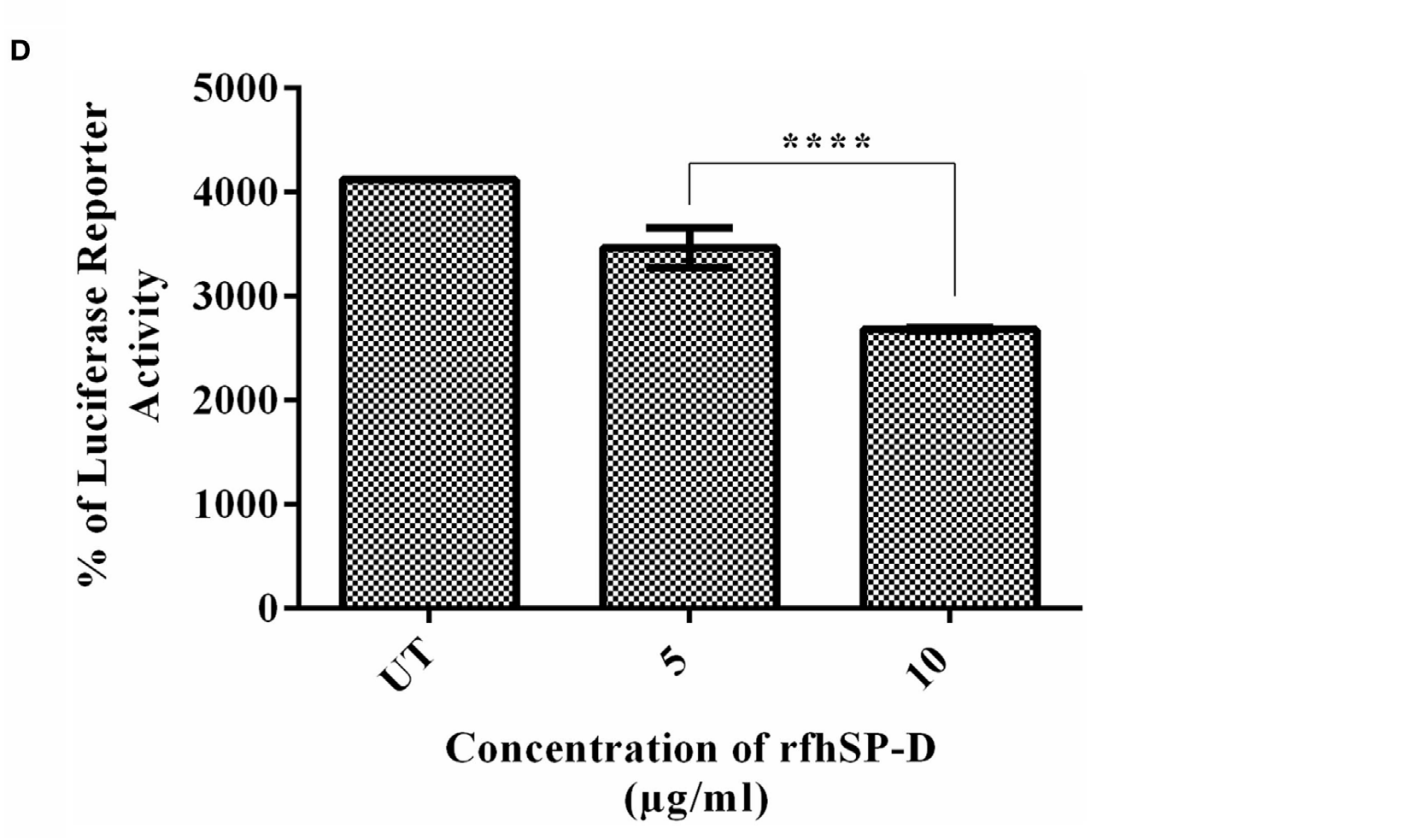
**(A)** Western blotting to show the expression of influenza A virus-hemagglutinin (HA) protein in purified H1+N1 pseudotyped lentiviral particles and cell lysate at 24 and 48 h. The presence of HA was identified at 70 kDa. **(B)** Far western blotting to show rfhSP-D binding in both purified H1+N1 pseudotyped lentiviral particles and cell lysate at 24 and 48 h. HA was evident at 70 kDa when probed with rfhSP-D. **(C)** Luciferase reporter activity of purified H1+N1 pseudotyped lentiviral particles at 24 and 48 h, and **(D)** Luciferase reporter activity of rfhSP-D treated MDCK cells transduced with these lentiviral particles. Significance was determined using the unpaired one-way ANOVA test (*p < 0.05 and ****p < 0.0001) (*n* = 3).

H1+N1 pseudotyped lentiviral particles were produced as a safe strategy to study the differential or combinatorial involvement of HA or NA viral glycoproteins in the recognition and neutralization of IAV by rfhSP-D. The production of lentiviral particles pseudotyped with envelope proteins H1+N1 was carried out by co-transfecting HEK293T cells with plasmid containing the coding sequence of the indicated H1+N1, pHIV-Luciferase backbone, and psPAX2. Purified H1+N1 pseudotyped particles and cell lysate harvested at 24 and 48 h were analyzed *via* western blotting, and the expression level of HA was determined using anti-H1 monoclonal antibody (Figure 8A); HA was evident at 70 kDa. Far western blotting revealed binding of rfhSP-D to HA at 70 kDa (Figure 8B), suggesting that the binding of rfhSP-D to HA is crucial for the inhibition of viral infectivity. Purified H1+N1 pseudotyped particles harvested at 24 and 48 h were used to transduce MDCK cells to measure the luciferase reporter activity assay. Higher levels of luciferase reporter activity were observed at 24 h when compared to 48 h post-transfection (Figure 8C). Thus, pseudotyped particles harvested at 24 h were used to transduce MDCK cells with or without rfhSP-D (5 and 10 µg/ml) (Figure 8D). Nearly 50% reduction in the luciferase reporter activity was observed with 10 µg/ml of rfhSP-D compared to cells challenged with H1+N1 pseudotyped particles. This suggested an entry inhibitory role of rfhSP-D against IAV.

## Discussion

Respiratory tract infection caused by IAV is associated with up to half a million mortality rates worldwide and five million cases of morbidity per year. A new swine-origin H1N1 IAV, identified in April 2009, spread worldwide, and was officially declared pandemic in June 2009. There are concerns that H1N1 or H3N2 viruses reassort with existing H5N1 virus using bird or pig as intermediate hosts, giving rise to more pathogenic IAV. Thus, it is important to understand molecular mechanisms of host’s first line of defense against IAV in order to design and develop novel and effective anti-IAV strategies. SP-D expressed at the mucosal sites including lungs plays an important role during IAV infection (22). SP-D has been shown to have a wide range of innate immune roles including neutralization, agglutination, opsonization and clearance of viruses including IAV. The binding ability of rfhSP-D to HIV-1 gp120 was reported, primarily in a dose- and calcium-dependent manner (23). Human SP-D has also been shown to bind IAV-HA and NA, resulting in the inhibition of viral attachment and entry into the host cells (24). However, the mechanism of direct inhibition of IAV and pseudotyped viral particles by SP-D and subsequent immune response is not fully explored.

Using two different IAV subtypes (pH1N1 and H3N2), we have shown that the entry inhibitory capability of rfhSP-D is not limited to a particular IAV subtype. To identify the interaction of rfhSP-D with IAV viral proteins, protein–protein interaction studies were carried out *via* ELISA, cell-binding assay, and far western blot. The ELISA (Figure 2) and cell-binding assay (Figure 3) revealed the maximal binding of rfhSP-D to both pH1N1 and H3N2 IAV subtypes at 5 µg/ml, and the maximum inhibition of cell binding was seen at 10 µg/ml of rfhSP-D. Furthermore, rfhSP-D bound purified recombinant HA protein in a concentration- and calcium-dependent manner (Figure 4C). rfhSP-D bound HA (70 kDa) and M1 (25 kDa) (Figure 4). N-linked oligosaccharides found on the IAV envelope glycoproteins (HA and NA) are known to be recognized by the CRD region of SP-D. Thus, HA-exposed glycans differing in location and numbers between IAV subtypes may be responsible for this interaction. rfhSP-D is likely to inhibit IAV infection by preventing the HA interaction with SA containing receptors. A reverse genetic approach has been used to analyze the role of N-glycosylation sites on the head of H1 in modulating sensitivity to SP-D *in vitro* and *in vivo* (25). It was found that HA Asn-144 was a critical factor in sensitivity to SP-D (25).

We also examined the immune response of A549 lung epithelial cells following IAV challenge in the presence or absence of rfhSP-D, which can impact upon cellular infection and viral replication. Therefore, the ability of rfhSP-D to modulate viral replication as well as inflammatory immune response following IAV challenge was examined *via* infection assay, qPCR, and multiplex cytokine array. The key aspect of host–pathogen interaction arising out of this study is the ability of rfhSP-D-bound pH1N1 and H3N2 to undergo suppressed replication, as evident by the expression of M1 gene. M1 is a matrix protein of IAV that lies beneath the lipid layer and is the most abundant protein, which is essential for viral stability and integrity. Thus, it plays a critical role in the recruitment and assembly of viral sites, nuclear export of viral RNPs, and thus, establishing the host components for viral budding (26). rfhSP-D suppressed the expression of M1 in pH1N1 (Figure 5A) at 2 h, while downregulating at 6 h in the case of H3N2 (Figure 5B). In addition, a lowered M1 expression was detected *via* western blot in the rfhSP-D treated sample compared to untreated sample following 6 h incubation (Figure 5C). Viral replication was also reduced in the presence of rfhSP-D as evident in Figures 5D,E. This suggests that rfhSP-D could act as an entry inhibitor against the subtypes tested (pH1N1 and H3N2). It is known that HA undergoes N-linked glycosylation, leading to modulation of antigenicity, fusion activity, receptor-binding specificity, and immune evasion of IAV. Therefore, SP-D can play an important role in innate defense against IAV as entry inhibitor by interfering with glycosylation sites and binding to glycans on the viral HA. It has been reported that the combinatorial substitutions of D325A/S+R343V in a trimeric neck and CRD fragment of human SP-D markedly increased anti-viral activity against pandemic IAV. This is because of the increased ability of the mutant to block the SA binding sites, aggregate the virus and reduce viral uptake (27).

Our qPCR data demonstrated an increased expression level of TNF-α and IL-6 in the case of H3N2 subtype (Figure 6B) compared to pH1N1. However, when pH1N1 was treated with rfhSP-D, TNF-α and IL-6 were suppressed at 2 h (Figure 6A), which recovered later (6 h). Elevated TNF-α and IL-6 levels can contribute to virus-mediated respiratory diseases or acute lung injury. IL-12 was considerably downregulated by rfhSP-D in the case of both IAV subtypes, suggesting the likely suppression of Th1 immune response. mRNA expression of RANTES was 10-fold (log_10_1 fold) downregulated in the presence of rfhSP-D at 2 h time point in pH1N1 compared to A549 cells challenged with IAV. However, cells, challenged with H3N2 and treated with rfhSP-D, were seen to have log_10_ one-fold downregulation of RANTES expression. In this study, we also report the ability of rfhSP-D (10 µg/ml) to downregulate both IFN-α and IFN-β expression (Figure 6C) at 2 and 6 h. Higher expression levels of IFN-α and IFN-β were detected in the untreated (cells + virus) sample, which was threefold (log10 fold) downregulated in the presence of rfhSP-D at 6 h. This suggests that when cells were incubated with pH1N1/H3N3 (MOI 1), higher levels of both IFN-α and IFN-β were produced by A549 cells to clear the virions. Since addition of rfhSP-D caused inhibition of viral replication, lower levels of INF were detected. Recently, the E3 ubiquitin ligase, TRIM29, has been shown to be a negative regulator of type I IFN responses in the lungs post-IAV challenge *in vivo*. TRIM29 acts by inhibiting IFN-regulatory factors and signaling *via* NF-κB, leading to degradation of NF-κB essential modulator (28). Whether rfhSP-D works *via* similar mechanisms is worth further investigation using SP-D gene-deficient mice (29). In addition, cytokine array analysis using supernatants that were collected at 24 h showed considerable downregulation of some of the key pro-inflammatory cytokines, chemokines, and other soluble factors in the presence of rfhSP-D. The downregulation of various humoral factors by rfhSP-D treatment could also facilitate the prevention of life-threatening secondary bacterial infections that may be caused by aberrant virus-mediated immune modulation.

In this study, we have produced the second-generation lentiviral vectors pseudotyped for H1+N1 of IAV. This system contains a single packaging plasmid (psPAX2) encoding genes including Gag, Pol, and Tat. pHIV-Luciferase was used as a lentiviral transfer plasmid, which is flanked with long terminal repeat (LTR) sequences, and designed to express the firefly luciferase reporter. Thus, pHIV-Luciferase is “replication incompetent” which contains an additional sequence deletion in the 3′ LTR leading to viral “self-inactivation” post-integration. This was selected as a safe alternative method to mimic the structure and surfaces of IAV, and to prove rfhSP-D as an entry inhibitor in cells transduced with pseudotyped IAV particles that are restricted to only one replicative cycle. The lentiviral particles pseudotyped with H1+N1 were analyzed *via* SDS-PAGE and western blotting. Expression of HA in purified H1+N1 pseudotyped lentiviral particles from transfected HEK293T cells was assessed by western blotting using anti-H1 monoclonal antibody (Figure 8A). H1+N1 pseudotyped lentiviral particles, purified *via* ultra-centrifugation, were used to investigate the combinatorial or differential involvement of viral envelope glycoproteins in the recognition and neutralization of HA by rfhSP-D. Incubation of rfhSP-D with these H1+N1 pseudotyped lentiviral particles was found to facilitate its binding to HA that appeared at 70 kDA in the far western blot (Figure 8B). To validate the effectiveness of rfhSP-D as an entry inhibitor of IAV, luciferase reporter activity assay was performed. Nearly 50% luminescent signal was seen with 10 µg/ml of rfhSP-D when compared to MDCK cells challenged with H1+N1 pseudotyped lentiviral particles alone. This, therefore, suggested the ability of rfhSP-D to inhibit viral infectivity through binding to cell surface bound HA found on the infected MDCK cells.

In summary, suppression of M1 expression, pro-inflammatory cytokine response, as well as luciferase reporter activity in target A549 cells by rfhSP-D highlight its potential as a therapeutic molecule in an entry inhibitory role against IAV.

## Author Contributions

MA-A, BN, and UK led the project; VM, PV, LK, and AP carried out crucial experiments; SA, IS, and AA-Q provided important reagents. VM, UK, and MA-A wrote the manuscript.

## Conflict of Interest Statement

The authors declare that the research was conducted in the absence of any commercial or financial relationships that could be construed as a potential conflict of interest.
